# General health and its relation to the quality of life and alcohol consumption in a Polish cohort of medical students – preliminary results of POLLEK survey

**DOI:** 10.3389/fpubh.2023.1178124

**Published:** 2023-07-03

**Authors:** Kamil Barański, Szymon Szemik, Angelina Kaleta-Pilarska, Małgorzata Kowalska

**Affiliations:** Department of Epidemiology, School of Medicine in Katowice, Medical University of Silesia in Katowice, Katowice, Poland

**Keywords:** GHQ28, audit, medical students, WHOQOL, health, alcohol consumption, quality of life

## Abstract

**Introduction:**

First-year students of medicine are at higher risk of stress related to the new environment and study overload. Such factors can play a role and have an impact on their quality of life and general health status which can cause possible problems with alcohol use. The aim of the study is to assess the relationship between mentioned factors in the Polish cohort of first-year medical students.

**Materials and methods:**

The quality of life has been assessed within the WHOQOL-BREF questionnaire, the general health status was assessed via GHQ28, and alcohol consumption was assessed the by AUDIT questionnaire. Due to the lack of signature informed consent and lack of data, 381 (72%) students out of 525 were included in the final analysis.

**Result:**

The majority of the 1-year students were females 68%; (*n* = 259) vs. 32% (*n* = 122) males. Half of the students had lowered risk of distress. However, females had a higher risk of having higher scores in GHQ-28 than males. In relation to the assessment of the quality of life, the students with a lower level of distress (<32 points in GHQ-28) had better results in each WHOQOL domain. There was no association between general health status and alcohol use. For the students who had possible problems with alcohol use the OR was 1.15 95% CI (0.73–1.80) and for students who were probably addicted OR was 1.07 95% CI (0.33–3.41).

**Conclusion:**

The total quality of life in first-year Polish medical students is relatively high; however, half of them suffer because of distress and around 30% have some alcohol problems. Females are more likely to have higher GHQ-28 scores than males.

## 1. Introduction

From 2015, the number of physicians with licenses to practice a profession in Poland increased from 141 360 to 155 800 in 2021, which gives an increase of ratio from 37.2 to 41 per 10 000 people, respectively (1). According to the OECD report: Health at a Glance: Europe 2020 Poland has one of the lowest numbers of practicing doctors (per 1000 people) in the whole European Union (2). Such a situation exposes medical doctors to an overload of duties. In consequence, medical doctors are at higher risk of sleep problems, alcohol/drug use, depression, burnout, etc. (3). Moreover, we observed a worrying trend of the outflow of medical staff from hospitals, which significantly worsening mentioned above problems in the public healthcare system and deepens the frustration of medical workers. The activities undertaken under METEOR project (4) have provided arguments that a particulary difficult situation is related to a Polish hospitals. The Meteor project is running to understand why healthcare workers leave their jobs, and to propose solutions that will improve their job satisfaction and working conditions. The most important factors that determine staff shortages in the whole country include disproportionately lower earnings, the sinister atmosphere at work, workload due to the lack of medical staff, no opportunity to prove competencies, no teamwork, bureaucracy, lack of patient care time, and dishonesty in the form of documenting procedures (5). In addition, it has been found that 9% of physicians wanted to migrate after the COVID-19 pandemic and 6% wanted to retire. One in ten nurses also wanted to either move abroad (3.8%) or retire (6.3%) (6). Less attention is paid to medical students. Current literature suggests that 25% of medical students are depressed, 18% are dependent on alcohol and 17.5% are burned out (7). Axiomatically, due to the considerable academic progression that medical students are exposed to during the transition from high school to the first year of medical school, they are at an increased risk of worsening physical, emotional, and overall health (8). Overall, this puts them into situations where they have to deal with psychological distress. They can use different coping methods to deal with problems. The coping mechanisms are very individual, but if the individual strategies are ineffective then it can lead them into burnout (9). Undetected general health problems, lowered quality of life, and alcohol addiction may persist into adulthood (10). Moreover, such aspects may have an impact on the intentions to leave their future workplace. Additionally, untreated mental issues may bring serious consequences such as impairment of their quality of life, increased risk of suicidal ideation, and decreased academic performance, professionalism and empathy toward their patients (11, 12). Our previous publication suggests that alcohol abuse among medical students is moderately widespread and implementation of screening programs in these groups is necessary (13). Recently, the law regulation regarding alcohol consumption in Poland has become more rigorous. People under age 18 cannot buy alcohol, alcohol cannot be consumed in public places, and local government can forbid the selling of alcohol between 22:00 and 6:00. However, a study on the mental health of medical students, which is also related to their quality of life level and alcohol consumption patterns, has not been carried out before in the Polish population. There is a need to bring new insight into how to improve the problematic situation in Polish hospitals by monitoring health and quality of life in future young physicians during their first year of medical studies.

Concerning the abovementioned arguments presented the study aimed to analyze the status of general health and its relation to alcohol consumption and quality of life in first-year students of medicine.

## 2. Materials and methods

There were 525 first-year students of medicine from the Medical Faculty in Katowice (Poland) invited to the study. The written consent was obtained from 433 students (67%; *n* = 292 females and 33%; *n* = 141 males), which resulted in a response rate of 82.5%. Due to a lack of data (no answers or incorrect fulfilling GOLDBERG (*n* = 25) or AUDIT (*n* = 26) questionnaire, 1 student did not fulfill both questionnaires) another 52 students were excluded from the study. Data from 381 students (68%; *n* = 259 females and 32%; *n* = 122 males) were obtained for final analysis.

The mean age of the students was 19.9 ± 1.8 years. Students were assessed with 3 questionnaires: World Health Organisation Quality of Life Brief Version (WHOQOL-BREF), Alcohol Use Disorders Identification Test (AUDIT), and General Health Questionnaire (GHQ-28). The full description of used methods (except GHQ-28) in our study is described in the previous publication (14). The project has the approval of the Bioethics Committee of the Medical University of Silesia in Katowice (approval number KNW/0022/KB/217/19; date: 08.11.2019).

The GHQ-28 questionnaire included questions related to 4 domains: depression (items 1–7), anxiety and insomnia (items 8–14), social dysfunction (items 15–21), and physical symptoms (items 22–28) (15). Analysis of this questionnaire results in a range score between 0 and 84 points. The recommended cut-off of GHQ-28 is >23 for being classified as psychiatric; however, the authors quoted publication suggests using the mean score as a cut-off. In our study, the median score from GHQ-28 was 31, and the mean score was 31.9. Because of the non-normally distributed points delivered from GHQ-28 in all students who participated in the study, we decided to use 31 points as a cut-off. The cut-off for specific domains was set according to a median score of a specific domain, 8 points for depression, 10 points for anxiety and insomnia, 8 points for social dysfunction, and 3 points for physical symptoms. The score below the cut-off (31 points) suggests a low level of distress. Moreover, students were asked about how they assess their current health conditions (self-assessment question) and if they are satisfied with their current health status (question from WHOQOL) to assess the relation to GHQ-28 scoring.

### 2.1. Statistical analysis

The quantitative variables were described as mean and standard deviation while the qualitative variables were described with a number (*n*) and frequency (%). The Shapiro–Wilk test was used to assess the distribution of quantitative variables. The differences between groups (GHQ ≤30 vs. GHQ >30 score) were analyzed with the t-students test or Wilcoxon test when appropriate. For qualitative variables the chi-square test was used. The association between variables: lowered risk of distress as the dependent variable and the alcohol addiction, and quality of life (good vs. bad) as an independent variable, was calculated with simple logistic regression and odds ratio (OR) with 95% confidence intervals. Simple linear regression (univariate model), and multivariate linear regression (coefficients with 95% confidence intervals were used for interpretation) were used to assess factors that influence the GHQ score. The following independent variables were considered in the model: sex, age, physical activity, smoking status, and occurrence of chronic disease. Spearman’s test was used to compute correlations between GHQ scores and Quality of Life scores. The level of significance in statistical analysis was set at a *p* < 0.05 value. All analyses were performed using SAS statistical package (SAS Institute Inc., Cary, NC, United Kingdom, version 9.4).

## 3. Results

A little more than half of the students (50.6%, *n* = 193) had lower levels of distress according to GHQ-28. Females had a more frequent lowered score of GHQ-28 (61%, *n* = 118) in comparison to males (39%, *n* = 75) and the observed difference was statistically significant (*p* = 0.003). The results of self-assessed health satisfaction and self-assessed health status questions were corresponding with GHQ-28 results because the lowered risk of distress was received by students who were assessing their health status as good or very good (77%, *n* = 149) or was satisfied with their health status (80%, *n* = 155). According to the quality of life assessed by WHOQOL, students who had lowered risk of distress had higher results in the total quality of life measurement and each domain in comparison to students who had increased values in GHQ. In the physical domain, the mean scoring was 48.7 in students who had lowered GHQ vs. 37.3 scoring in students who had higher GHQ, for the psychological domain 66.0 vs. 55.2 points, in the social domain 75.5 vs. 63.5 points, and in environmental domain 67.9 vs. 60.1 points, respectively. All differences were statistically significant, detailed data were presented in the Table 1.

**Table 1:** Sex, age, marital status, and quality of life according to general self-declared health status in all participants of the study.

Variable		GHQ score 28 item		*p* value
		Lower GHQ score ≤ 31 N = 193; 50.6%	Higher GHQ > score 31 N = 188; 49.4%	
Sex n; %	Male Female	75; 38.9% 118; 61.1%	47; 25.0% 141; 75.0%	0.003
Age X; SD		20.0 ± 1.90	19.9 ± 1.72	0.6
Marital status	In relationship Single	45; 23.7% 145; 76.3%	45; 24.5% 139; 75.5%	0.8
Current health condition (self-declared)	Bad or very bad Neither bad nor good Good or very good	8; 4.1% 36 18.7% 149 77.2%	28; 14.9% 81; 43.1% 79; 42.0%	<0.0001
How satisfied are you with your health? (WHOQOL-BREF)	Very dissatisfied or dissatisfied Neither satisfied nor dissatisfied Satisfied or very satisfied	14; 7.3% 23; 12.0% 155; 80.7%	39; 20.7% 56; 29.8% 93; 49.5%	<0.0001
QOL-BREF		75.0 ± 15.1	61.0 ± 17.9	<0.0001
QOL Physical health domain		48.7 ± 10.6	37.3 ± 12.2	<0.0001
QOL Psychological domain		66.0 ± 10.1	55.2 ± 12.0	<0.0001
QOL social relationship domain		75.5 ± 15.4	63.5 ± 18.8	<0.0001
QOL environmental domain		67.9 ± 12.2	60.1 ± 11.6	<0.0001
AUDIT scoring N;%	<8 ≥8 and ≤ 14 ≥15	134; 69.4% 55; 28.5% 4; 2.1%	125; 66.5% 59; 31.4% 4; 2.1%	0.5

### 3.1. The relationship between self-declared general health, quality of life, and alcohol consumption

In the whole study group, there were 16.2% (*n* = 62) students who had a higher risk of distress (>31 points) general health score, lowered health quality (<60 points), and possible problems with alcohol consumption (>7 points). Five students obtained AUDIT scoring suggested addiction to alcohol, had lowered their quality of life, and had a higher risk of distress.

**Figure 1:**
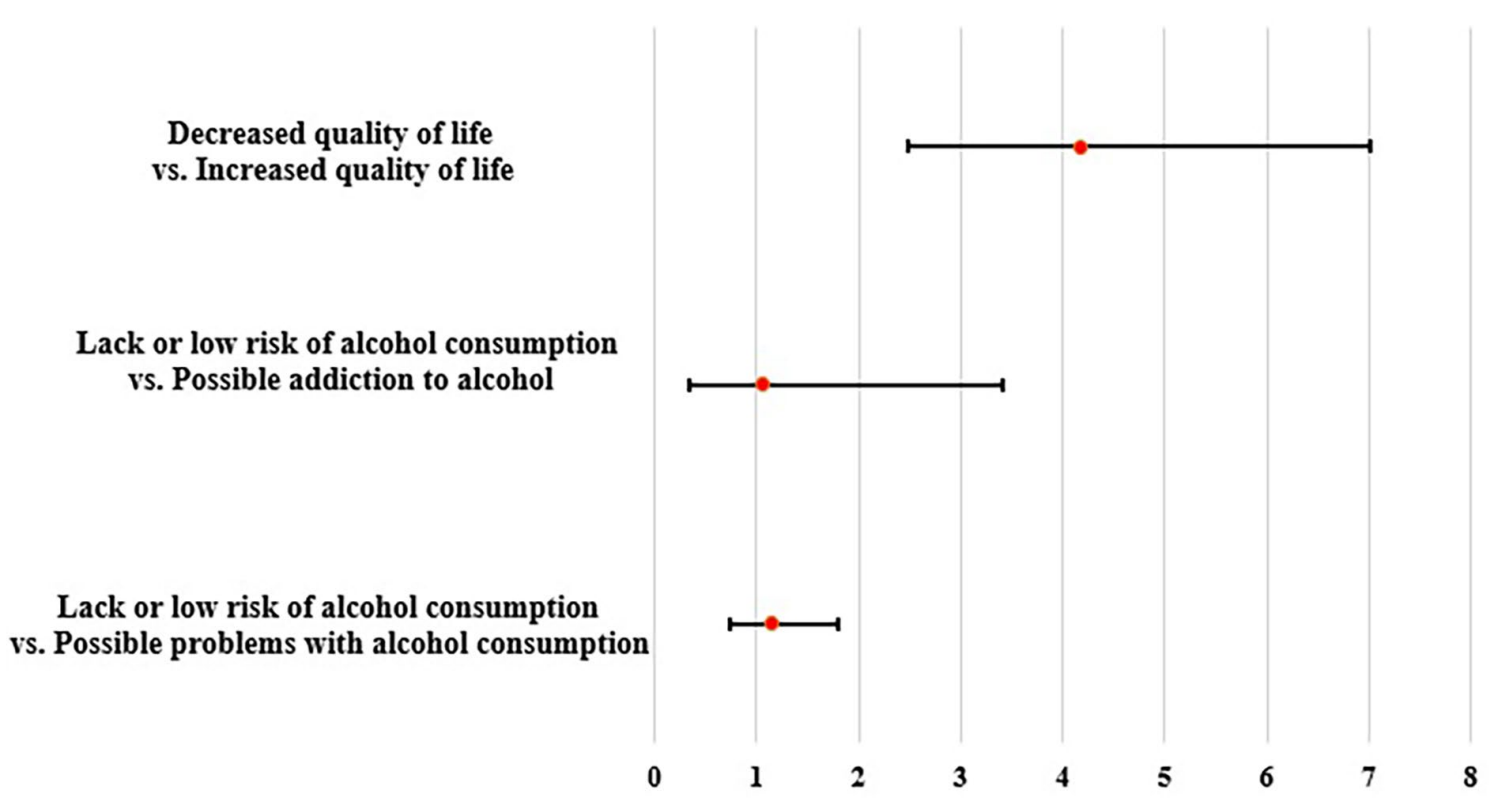
Factors influencing the reduced risk of distress in first-year medical students.

There was no association between alcohol use and lowered general health. For students who had possible problems with alcohol consumption the risk of lowered general health was OR = 1.15 95%CI (0.73–1.80) and in students who were probably addicted OR = 1.07 95% CI (0.33–3.41) (Figure 1). In relation to the quality of life, students who had lowered quality of life had a higher risk of having increased level distress OR = 4.17 95% CI (2.48–7.02) (Figure 1).

The mean score for depression was 8.5 ± 3.7 points, for anxiety and insomnia 10.0 ± 4.5 points, for social dysfunction 9.0 ± 3.4 points, and physical symptoms 4.2 ± 4.4 points. From all students, 50.1% (*n* = 191) had increased scores of depression, 46.9% (179) had increased scores of insomnia and anxiety, 52.5% (*n* = 200) had increased scores of social dysfunction, and 43.0% (*n* = 200) had increased scores of physical symptoms. Students, with lowered quality of life, had a higher risk of depression OR = 3.73 95% CI (2.23–6.24), anxiety and insomnia OR = 2.64 95% CI (1.63–4.29), social dysfunctions OR = 2.36 95% CI (1.44–3.87) and physical symptoms OR = 3.74 95% CI (2.28–6.13). In relation to alcohol consumption, there was no significant association with declared general health. The odds ratios were 0.99, 1.27, 1.03, and 1.24, respectively. It is worth noting that every third student (34.6%, *n* = 212) declared a lack of any health and quality of life issues.

### 3.2. Factors related to worse general health status

The result of the analysis showed that students who never smoked before had the lowest score of GHQ-28 30.4 ± 12.5, followed by past smokers 32.0 ± 12.2, and current smokers 36.3 ± 12.7 (*p* = 0.008). When considering students with a lower risk of distress (GHQ-28 ≤ 31 points), the frequency of lower risk of distress was 55.6% (*n* = 114) for never-smokers, 47.7% (*n* = 54) past smokers, and 40.3% (*n* = 25) current smokers (*p* = 0.08). The mean score of GHQ-28 value in students who declare physical activity not less than 30 min 3 times per week was 28.5 ± 11.9, for less active students it was 32.2 ± 11.7 and for students, without any physical activity, it was 36.1 ± 14.6 points (*p* = 0.003). When considering GHQ-28 cut-ff the frequency of lowered risk of distress GHQ-28 scores was 58.7% (*n* = 57) in physically active students, 49.3% (*n* = 106) who were less physically active, and 42.6% (*n* = 29) for students without any physical activity (*p* = 0.1).

The significant variable which differed GHQ-28 scores between students was the occurrence of chronic disease. Students who had chronic disease ever diagnosed by a physician had 35.0 ± 12.5 points in GHQ-28 and students without any chronic disease had 30.9 ± 12.5 points (*p* = 0.01). When considering the GHQ cut-off, the lowered risk of distress was found in 42.5% (*n* = 37) students with chronic disease and 53.4% (*n* = 156) students without any chronic disease.

The analysis included, as well correlation between the GHQ-28 score and the score of the WHOQOL-BREF questionnaire. The quality of life scale was negatively associated with GHQ-28 score R = −0.50; *p* < 0.0001.

Univariate analysis has detected 5 variables associated with GHQ-28 scores (Table 2). Further multivariate analysis identified two variables associated with the GHQ-28 score, they were sex and quality of life assessed by the WHOQOL-BREF questionnaire (Table 2).

**Table 2:** Results of univariate and multivariate linear regression analysis for prediction of general health questionnaire score.

Variable	Value	Univariate	Multivariate	Model
		*p* value	Coefficient (95% CI)	*p* value
Sex	Male	<0.0001	reference	0.001
	Female		3.83 (1.48–6.18)	
Quality of life	Continuous variable	<0.0001	−0.32 (−0.39–0.26)	<0.0001
Smoking status	Never	0.002	−1.66 (−4.84–1.51)	0.5
	In the past		−1.56 (−4.94–1.82)	
	Current smoker		reference	
Physical activity	Yes, 3 times 30 min per week	0.001	−3.29 (−6.75–0.15)	0.1
	Yes, not so often		−1.19 (−4.20–1.82)	
	Never		reference	
Chronic disease diagnosed by a physician	Yes	0.008	0.47 (−2.17–3.12)	0.7
	No		reference	

## 4. Discussion

Our study assessed the relationship between the results of the General Health Questionnaire and the results of the Quality of Life measured by the QOL-WHO Breef questionnaire and alcohol use measured by the AUDIT questionnaire. The first doubts related to the results of the study were the assessment of association and direction between those three variables. It seems that those variables influence each other mutually. However, according to the results of our study, the level of distress is not associated with alcohol use. This finding was not consistent with the results of the study performed on the general Finnish population where the AUDIT score was associated with poorer mental health; however, this might be due to different assessment tools with fewer questions. The authors of the mentioned paper used a shorter version of GHQ (16). Other sources suggest that GHQ-12 is not a recommended screening tool for routine use (17).

Moreover, in the other study, the authors measured the risk of distress with GHQ-12 and addiction to alcohol by CAGE (Substance Abuse Screening Tool). The correlation between the results of both tools was nonsignificant and very weak (R = 0.083, *p* = 0.2) (18). However, alcohol use with other analyzed factors in a group of students might be affected by the student’s lifestyle, where consumption of alcohol increases because of sociological impact (19). The higher alcohol consumption is related to the male sex (13). In Poland, the factors that predispose young males to drink alcohol are not much different from the other countries. Among the others, drinking motivated by social reasons, to celebrate, to have a good time, or to enhance one’s social confidence on the one hand, and drinking to cope (negative emotions), to escape, or to avoid or regulate unpleasant emotions on the other (20).

In our study, males, in comparison to females, had more often possible problems with alcohol, 43% vs. 26%, respectively. The level of drinking alcohol by Polish medical students is higher in comparison to other nations like Germany or Thailand (21, 22). Some reports suggest that medical students are more prone to alcohol abuse than the non-medical students. The risk factors of alcohol abuse were younger age, being single, and higher educational debt in the medical students according to results of Shah study (23). In the United Kingdom based students, BMJ (British Medical Journal) subscribers, one in 10 medical students exceeds weekly alcohol consumption (24). On the other hand, the conflicting results reported by researchers from Sweden who shown the harmful alcohol use in 38.5% (*n* = 70) medical students and 60.4 (*n* = 106) business students (25).

The quality of health was measured with the commonly used method in other studies. The biggest issue related to WHOQOL-BREF use is its interpretation. For our study, we used a suggested cut-off of <60 points (26). In our study, 25% (*n* = 94) of students declared their quality of life as lowered. The results from the specific domains are high (before adjustment according to WHOQOL instructions) (27) in comparison to the general young population students from our study had higher quality of life in physical health (a mean value of 19.0 vs. 14.4 points), psychological health (mean value of 20.5 vs. 13.3 points), environment (mean value of 28.5 vs. 12.9 points) except for social relationships (mean value of14.0 vs. 11.3 points) (28). When comparing satisfaction from health status assessed by WHOQOL it seems that our study group either was more satisfied with health quality (mean value of 3.63 vs. 3.21 points) (28). Our results are different than the observation of Messina et. all in which the quality of life of medical students was lower in comparison to the general population (29). Moreover, in our study, the were no differences between sexes according to the total quality of life. More than 70% (*n* = 188) of females and 81% (*n* = 99) of males had a total quality of life qualified as good;, this result is consistent with the finding of another researcher (30). However, it seems that males better evaluate their quality of life status. The difference between sexes was found in a study performed in Taiwan, where in most domains males had higher scores (31). In relation to general health status assessed by GHQ-28 in medical students, the female sex was associated with a higher risk of having distress in comparison to males, this finding was similar in other studies (32, 33). Unfortunately, half of the students are struggling because of distress; in our study it concerned 49.3% of the students, while in the study conducted by Jafari, it was 49.5% of students. Such results are confirmed in a meta-analysis performed among Chinese medical students (34).

### 4.1. Limitations

Our study has some limitations. The first issue is related to the methods used in the study. All data about the quality of life, alcohol use habits, or level of distress are self-reported. However, we used standardized questionnaires which should improve the reliability of the results. Another study limitation is the sample size. In comparison to other studies, we could achieve more consistent results if we could run another edition of our study. Moreover, the participants are representative probably only for Silesian Voivodship in Poland, because most of the students are from the Silesia region, but some of the are from other parts of Poland. The cross-sectional nature of the study measured only at one point does not allow to make a conclusion about chronic effects of alcohol consumption and distress in medical students. Moreover, in our study, there was a big disproportion between sexes, such situation could create a possible confounding issue, we controlled that effect with multivariate effect. Moreover, the feminisation of medical school has been observed previously and it is still an observable situation (35). The strength of the study is that we simultaneously analyzed both the quality of life, general health status, and habits related to alcohol consumption in a similar group of respondents; first-year medical students. It is an important step in the follow-up type of study in which we want to evaluate the baseline situation in case of the health status and behaviours of future physicians.

## 5. Conclusion

The total quality of life in first-year Polish medical students is relatively high; however, half of them suffer because of distress and around 30% have some alcohol problems. It seems important to continue supporting young people starting their studies at a medical university in terms of maintaining a healthy lifestyle and coping with stress.

## Data availability statement

The original contributions presented in the study are included in the article/Supplementary material, further inquiries can be directed to the corresponding author.

## Ethics statement

The studies involving human participants were reviewed and approved by Komisja Bioetyczna Śląskiego Uniwersytetu Medycznego w Katowicach. The patients/participants provided their written informed consent to participate in this study.

## Author contributions

MK contributed to the concept and design of the study. SS and MK obtained data for work. KB and MK prepared a database and conducted a statistical analysis. KB wrote the manuscript. The AK-P edited the work. All authors contributed to the article and approved the submitted version.

## Funding

The project received financial support from the Medical University of Silesia (grant number: PCN-1-047/K/1/0).

## Conflict of interest

The authors declare that the research was conducted in the absence of any commercial or financial relationships that could be construed as a potential conflict of interest.

## Publisher’s note

All claims expressed in this article are solely those of the authors and do not necessarily represent those of their affiliated organizations, or those of the publisher, the editors and the reviewers. Any product that may be evaluated in this article, or claim that may be made by its manufacturer, is not guaranteed or endorsed by the publisher.
